# Hepatic Macrophage Responses in Inflammation, a Function of Plasticity, Heterogeneity or Both?

**DOI:** 10.3389/fimmu.2021.690813

**Published:** 2021-06-09

**Authors:** Christian Zwicker, Anna Bujko, Charlotte L. Scott

**Affiliations:** ^1^ Laboratory of Myeloid Cell Biology in Tissue Damage and Inflammation, VIB-UGent Center for Inflammation Research, Ghent, Belgium; ^2^ Department of Biomedical Molecular Biology, Faculty of Science, Ghent University, Ghent, Belgium; ^3^ Department of Chemical Sciences, Bernal Institute, University of Limerick, Limerick, Ireland

**Keywords:** hepatic macrophages, Kupffer cells, inflammation, infection, recruited macrophages, liver, myeloid cells

## Abstract

With the increasing availability and accessibility of single cell technologies, much attention has been given to delineating the specific populations of cells present in any given tissue. In recent years, hepatic macrophage heterogeneity has also begun to be examined using these strategies. While previously any macrophage in the liver was considered to be a Kupffer cell (KC), several studies have recently revealed the presence of distinct subsets of hepatic macrophages, including those distinct from KCs both under homeostatic and non-homeostatic conditions. This heterogeneity has brought the concept of macrophage plasticity into question. Are KCs really as plastic as once thought, being capable of responding efficiently and specifically to any given stimuli? Or are the differential responses observed from hepatic macrophages in distinct settings due to the presence of multiple subsets of these cells? With these questions in mind, here we examine what is currently understood regarding hepatic macrophage heterogeneity in mouse and human and examine the role of heterogeneity *vs* plasticity in regards to hepatic macrophage responses in settings of both pathogen-induced and sterile inflammation.

## Introduction

Macrophages, first described by Ilya Metchnikoff over 100 years ago (1), are widely recognised as key players of the innate immune system. Macrophages are present in almost every tissue of the body where they function to sense their local environment and to clear pathogens and debris including dying cells. As such, macrophages are thought to be a considerably plastic cell population, able to rapidly respond to changes in the tissue environment and to assume different cellular phenotypes as required (2). This idea stems from findings that both *in vitro* bone-marrow (BM) macrophages and *in vivo* macrophage populations respond differently depending on the specific stimuli they sense in their local environment. While the plasticity of *in vitro* generated BM-derived macrophages is uncontested, the recent technological advances including multi-parameter flow cytometry/mass cytometry and single cell RNA sequencing (scRNA-seq) have demonstrated considerable heterogeneity within the macrophage pool of different tissues, including the liver, particularly under non-homeostatic conditions (3–8). In inflammation a population of monocytes are recruited to the tissue, where they then locally differentiate into macrophages (9, 10), which accompany the already present tissue-resident macrophages. This has, therefore, brought the concept of macrophage plasticity *in vivo* into question; is the perceived plasticity of macrophages rather due to the presence of different macrophage populations with distinct functions? Moreover, while these recruited macrophages have been shown to be able to take on a wide range of phenotypes (3, 4, 11, 12) other recent studies have suggested that tissue-resident macrophages (13, 14) may not respond as extensively to insults as previously thought (4, 13–16). As the origins of tissue-resident (primarily embryonic) and recruited macrophages (BM-derived) differ (17–19), this also brings the role of ontogeny into question when investigating macrophage plasticity (18). However, whether macrophage plasticity would be inherently linked to ontogeny or rather the length of time a macrophage spends in a specific tissue environment or niche, where it is being continually instructed by the other cells in that niche (20) remains an open question.

Understanding macrophage plasticity is of crucial importance not only to better understand macrophage fate and functions but also in the design of therapeutic approaches aimed at manipulating macrophage function. For example, a plastic macrophage might represent a more amenable target in clinical settings. Alternatively, if not plastic, the strategy to replace the macrophage population may be more beneficial. In this review, we thus aim to provide our viewpoint on the role of macrophage heterogeneity *vs* plasticity in the context of the liver. First, we will provide an overview of the current state-of-the-art regarding the different macrophage populations that have been described across different settings in the murine and human liver. Subsequently, we will discuss what is known, alongside the remaining questions regarding the specific roles of the distinct hepatic macrophage populations and their associated plasticity in the context of both pathogen-induced and sterile inflammation.

## Hepatic Macrophage Populations

Before we can investigate functions and potential plasticity of hepatic macrophages in sterile and pathogen-induced inflammation, we must first consider the different macrophage populations present in the liver under different settings. Thus, here we will first introduce the main subsets present across species and inflammatory settings and the terminology by which we will refer to them throughout this review.

### Murine Hepatic Macrophages

**Figure 1:**
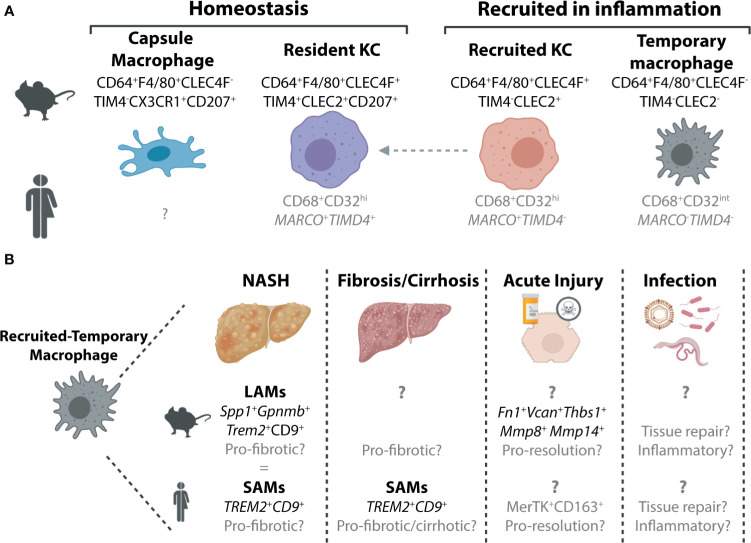
Murine and Human Macrophage Populations in the Healthy and Inflamed Liver. **(A)** Two distinct populations of hepatic macrophages have been defined to date in the murine liver, the resident KCs, which make up the majority of the hepatic macrophage population and a smaller population of macrophages found in the liver capsule (21). In inflammation, these populations are often accompanied by populations of recruited macrophages. These can also exist in at least two subsets, recruited KCs which can persist in the tissue to generate resident KCs (4, 22–26) and a population of macrophages that are lost from the liver upon resolution of inflammation (15) here termed recruited-temporary macrophages. In the human liver, we still do not fully understand the distinct populations of macrophages present and how these relate to those found in mice. To date no counterpart for the murine capsule macrophages has been identified, however, this may be due to difficulties in isolating cells from the capsule, particularly from smaller liver biopsies which do not harbour significant amounts of capsule tissue. While all macrophages in the human liver can be identified on the basis of their expression of CD68, scRNA-seq studies have revealed that these can be further split into distinct subsets (27–30). Two populations of macrophages have been identified in healthy human liver tissue, which are distinguished by their expression of *MARCO* and *TIMD4*. Here we speculate (shown in grey) that the *MARCO* ^+^ *TIMD4* ^+^ cells would be the counterparts of the murine resident KCs while the cells lacking expression of these two genes could be considered as recruited-temporary macrophages. In inflammation, a population of *MARCO* ^+^ *TIMD4* ^-^ macrophages has also been identified thus it is tempting to align these with murine recruited KCs (30), however this also requires validation. In addition to these genes delineating subsets of human hepatic macrophages, CD32 has also recently been suggested to be a good protein marker to distinguish between these macrophages (31). **(B)** Due to their recent identification within the hepatic macrophage pool, the precise nature of the recruited-temporary macrophage population also remains unclear. In certain inflammatory settings, such as NASH and fibrosis/cirrhosis, it has recently been shown that these cells express genes including *Spp1, Gpnmb* (mouse) *Trem2* and *Cd9* (mouse and human). In mouse, these cells were termed lipid-associated macrophages (LAMs) while in human they were called scar-associated macrophages (SAMs) (4, 30, 32). Alignment of the LAMs and SAMs showed significant overlap (4) suggesting these could indeed be equivalent populations. In other inflammatory settings much less is known about these cells, and hence it is unclear if these cells also have a LAM/SAM phenotype or if their phenotype is dependent on the inflammatory stimulus. In acute liver injury in mice, they have been suggested to express genes associated with a function in resolution as well as genes associated with extracellular matrix (15). In human acute liver injury, a population of MerTK^+^CD163^+^ macrophages have been reported which may represent human recruited-temporary macrophages (33). Moreover, to date, the specific functions of these cells are largely speculative. To date, limited data is available regarding recruited-temporary macrophages in infection in mice and humans and thus their potential function(s) remain speculative.

To date, the homeostatic murine liver has been demonstrated to harbour two distinct macrophage populations, Kupffer cells and capsule macrophages (**Figure 1**). Kupffer cells (KCs), first described by Karl Wilhelm von Kupffer (34), are the resident macrophages of the liver, which represent one of the most abundant macrophage populations in the body. These cells, defined by their expression of CLEC4F, TIM4, F4/80 and CD64 (22, 35) (**Figure 1**), reside in the liver sinusoids where they further extend a proportion of their body to contact hepatic stellate cells (HSCs) and hepatocytes (36). KCs are largely found throughout the liver, however, are relatively sparse in the immediate vicinity of the central vein (37). As has been reviewed extensively, KCs, are long-lived, self-renewing macrophages, that exist, at least in the mouse, largely in the absence of any input from circulating BM (17, 19, 20). The one exception to this is a short time window in the first weeks of life, when the liver is growing (22). On the other hand, liver capsule macrophages reside, as their name would suggest, in the liver capsule (21). Here they express CD64 and F4/80, but lack expression of CLEC4F and TIM4. Capsule macrophages are also identified through their expression of CX3CR1 (absent on KCs) and CD207 (langerin; also expressed by KCs) (21) (**Figure 1**). Unlike KCs, capsule macrophages are relatively short-lived and arise primarily from monocytes (21).

While relatively straight forward under homeostatic conditions, in inflammation, the murine hepatic macrophage pool becomes considerably more complex. Inflammation is often linked to a reduction in the resident KC pool (4, 15, 23–25, 32, 38, 39). However, while in some studies this has been demonstrated numerically (4, 25, 32, 38, 39), in others this drop in resident KCs is observed only as a reduction in proportion. This may result from an increase in other recruited cells (e.g., monocytes, neutrophils) rather than a drop in numbers of resident KCs per se (15, 23, 24). Whether in number or proportion, this reduction in resident KCs is linked to the recruitment of monocytes (Ly6C^hi^, CD11b^+^, F4/80^-^, CD64^lo/int^), which rapidly differentiate into hepatic macrophages (Ly6C^-^, CD11b^-/+^, F4/80^+^, CD64^+^). Under some conditions, these recruited macrophages can further differentiate into recruited KCs which can be temporally distinguished from resident KCs based on their lack of TIM4 expression (4, 22–26, 32, 40) (**Figure 1**). As recruited KCs can acquire TIM4 expression with time (22) (**Figure 1**), long-term (>1month) tracking of these cells is only possible through the use of fate-mapping methods such as BM chimeras, parabiosis or genetically-labelled mouse models (22). However, the use of such fate-mapping methods can also impact the results. For example, the irradiation required to generate BM chimeras may further injure the liver, rendering results difficult to interpret. Irradiation may also alter disease progression in some models. With this in mind, many studies utilise flow cytometric methods instead which are often quicker and less invasive. However, without a permanent marker of origin, these studies cannot distinguish recruited KCs on the long-term. Whether a distinction between resident and recruited KCs is required or is rather an issue of semantics remains debated. Recruited KCs have been shown to acquire the full transcriptional profile of resident KCs, when generated following resident KC depletion using Diptheria toxin administration in *Clec4f-*DTR mice (22). This ability to acquire the resident KC profile is driven by the imprinting of differentiating monocytes by the cells of the local KC niche including liver sinusoidal endothelial cells (LSECs), HSCs and hepatocytes (36, 41, 42). However, whether recruited KCs developing in an inflamed environment would receive the same imprinting and hence resemble the steady-state resident KC counterparts remains unclear. Moving forward, the recent generation of new genetic mouse models that allow monocyte-progeny to be fate mapped will help to resolve some of these questions (43).

In addition to generating KCs, recruited monocytes can also have a different fate, differentiating into macrophages distinct from KCs (4, 15, 32). In many models where inflammation is resolved including acetaminophen (paracetamol; APAP) overdose induced acute liver injury, these recruited non-KC macrophages appear to be short-lived and lost upon resolution of inflammation (15). Thus, from herein we will refer to these cells as recruited-temporary macrophages (**Figure 1**). As many studies have not investigated potential macrophage heterogeneity, the distinction between resident KCs, recruited KCs and recruited-temporary macrophages is often not clear and hence it remains to be seen if all these cells are a common feature of liver inflammation. Moreover, capsule macrophage responses in inflammation have not yet been assessed. Additionally, for many settings we still lack specific markers for the recruited-temporary macrophage population, making it difficult to distinguish these cells from capsule and recruited macrophages on their way to becoming KCs, prior to their acquisition of KC markers such as CLEC4F, which takes ~7 days (22) (**Figure 1**). Recently, CLEC2 has been proposed to be an early marker of recruited KCs as expression is observed before cells become CLEC4F^+^ (4, 23). However, without more specific markers of the remaining CLEC2^-^ cells it remains difficult to identify the *bona fide* recruited-temporary macrophage population(s) that may be present (**Figure 1**). It should also be noted that while here we refer to these cells as recruited-temporary macrophages, in chronic models, it is unclear if these cells are (a) temporary and hence lost upon resolution of inflammation or (b) continuously replaced or persistent throughout the course of inflammation. Given the potential role for local niche signals in imprinting macrophage phenotype as is observed for KCs, we would hypothesize that recruited-temporary macrophages would be lost upon return to homeostasis as the niche inflammation is also resolved, hence their designation as temporary, however this remains to be tested. In metabolic-associated fatty liver disease (MAFLD), a population of recruited-temporary macrophages distinct from KCs and capsule macrophages called lipid associated macrophages (LAMs) has been identified (4) (**Figure 1**). These are proposed to develop through an intermediate population termed c-LAMs (32). LAMs and c-LAMs were found to preferentially localise in zones of fibrotic tissue and had a transcriptional profile distinct from resident and recruited KCs (4, 32). While the size of this population increased with time on the diet, correlating with both weight gain and the degree of fibrosis (4), whether this increase is due to local proliferation or continuous engraftment has not yet been investigated. Moreover, the fate of these cells upon return to normal chow has not yet been studied. Taken together, in inflammation the murine liver can harbour at least 4 distinct populations of macrophages, resident KCs, recruited KCs, capsule macrophages and recruited-temporary macrophages (**Figure 1**). Notably, aside from the proposal of niche availability (20), the precise micro-environmental cues determining the fate of recruited monocytes remain unclear (see below). Additionally, the degree of homogeneity within the recruited-temporary macrophage pool across inflammatory conditions remains to be investigated.

### Human Hepatic Macrophages

As humans do not live in specific pathogen free (SPF) environments with restricted diets, exactly how comparable and relevant the homeostatic mouse hepatic macrophage populations are to those observed in the healthy human liver is debatable. Despite this, scRNA-seq and single nuclei sequencing (snRNA-seq) of healthy caudate lobes of the human liver (non-transplanted tissue) have identified two distinct populations of hepatic macrophages (27, 28) (**Figure 1**). As both expressed CD68 and were found in the healthy liver, they were both termed KCs. *CD68* ^+^ *MARCO* ^+^ KCs appear to closely resemble murine resident KCs in terms of their transcriptome (27). In addition to transcriptional similarities, like murine resident KCs, MARCO^+^ macrophages were also largely absent from peri-central regions of liver tissue, instead predominating in peri-portal regions as assessed using immunohistochemistry (27). Thus, *MARCO* ^+^ KCs may be *bona fide* human resident KCs, although identification of additional markers expressed by these cells and conserved with murine resident KCs would help to support this conclusion. In addition to the putative human resident KCs, *CD68* ^+^ *MARCO* ^-^ KCs were also identified in the healthy human liver. These cells were enriched for genes associated with monocytes including *FCN1, S100A8* and *LYZ* and showed an increased propensity for TNFα production following stimulation with LPS and IFNγ *ex vivo* (27). As such, these were designated as inflammatory KCs. Similarly, another scRNA-seq study also reported the presence of two KC populations in the healthy human liver (29), while a third study, using flow cytometry, suggested CD32 to be a useful surface marker to discriminate between these two KC subsets (31). However, as these latter two studies utilised normal tissue adjacent to resected tumour tissue as healthy liver tissue, how representative these macrophage populations are of those found in healthy livers remains unclear. Notably, the CD32^int^ subset identified as the inflammatory KC population also expressed CD1c (29, 31) which is a typical marker associated with human conventional type 2 Dendritic cells (cDC2s) (44–46). Thus, the designation of these cells as KCs clearly requires further validation, as this could potentially represent a mixed population of myeloid cells. Indeed, as KCs defined in these 3 studies were identified as such primarily based on their expression of CD68 and their presence in the healthy liver, it will be important in general to validate the claim of KC identity. For example, can we be sure that these are both KCs and one population does not represent human capsule macrophages as observed in the murine healthy liver? Alternatively, as even a healthy human will likely have experienced infection, eaten fatty food or consumed alcohol, another possibility is that these inflammatory KCs are equivalents of the murine recruited-temporary macrophages (**Figure 1**). This is especially likely when healthy liver tissue is obtained from patients undergoing liver resection due to cancer metastasis (29, 31). Suggesting that these *MARCO* ^-^ macrophages may indeed be recruited-temporary macrophages rather than KCs, a recent study by the Henderson lab investigating myeloid cell heterogeneity in healthy and cirrhotic human livers found *MARCO* ^-^ *CD68* ^+^ macrophages to be enriched in cirrhotic tissue (30). Consistent with this, *Marco* expression in the mouse, was also restricted to KCs in the fatty murine liver (4), although whether this holds true in other inflammatory settings remains to be investigated. In addition, this study also identified additional heterogeneity within the *MARCO*-expressing KCs (30). Here *MARCO* ^+^ KCs in the healthy human liver (resected material) could be further divided into two populations, one expressing *TIMD4* and one lacking expression of *TIMD4* reminiscent of the murine resident and recently recruited KC populations respectively (30) (**Figure 1**).

Consistent with the murine liver, in inflammation, there is also further heterogeneity within the human hepatic macrophage pool. In the cirrhotic liver, similar to the inflamed murine liver, a reduction in the proportion of resident KCs (*MARCO* ^+^ *TIMD4* ^+^) was observed (30). This was accompanied by an increase in the proportion of *CD68* ^+^ *MARCO* ^-^ macrophages expressing *CD9* and *TREM2* which were termed Scar-associated macrophages or SAMs due to their proximity to cirrhotic scar tissue (30). Thus, SAMs are likely a subset of recruited-temporary macrophages in the human liver. While the SAMs described here share some overlap with the inflammatory KCs described above including *FCN1, CD68* and *LYZ* expression (27, 30), the precise overlap between these cells requires further investigation (**Figure 1**). Notably, these SAMs are also reminiscent of LAMs, the recruited-temporary macrophages recruited to the liver in murine models of fatty liver disease (4) (**Figure 1**). Similar to LAMs, the temporal nature of these SAMs remains to be established. In addition to SAMs, a population of macrophages expressing MerTK and preferentially localised around centrilobular necrosis in the livers of patients following APAP overdose have been described (33). As MerTK expression was relatively sparse in the rest of the liver this could suggest that these cells are a population of recruited-temporary macrophages (**Figure 1**), indeed this would be consistent with the increased expression of MerTK observed in circulating blood monocytes of these patients compared with healthy controls (33). Alternatively, resident KCs may upregulate MerTK expression upon APAP overdose, as secretory leucocyte protease inhibitor (SLPI), produced in acute liver failure, was shown to induce MerTK expression in monocytes and hepatic macrophages (33). Importantly, exactly how similar these cells are to the hepatic macrophage populations described in the single cell studies remains to be seen. Thus, taken together, it is clear further work is required to fully understand human hepatic macrophage heterogeneity in health and disease.

## Hepatic Macrophage Responses: A Function of Plasticity or Heterogeneity?

**Figure 2:**
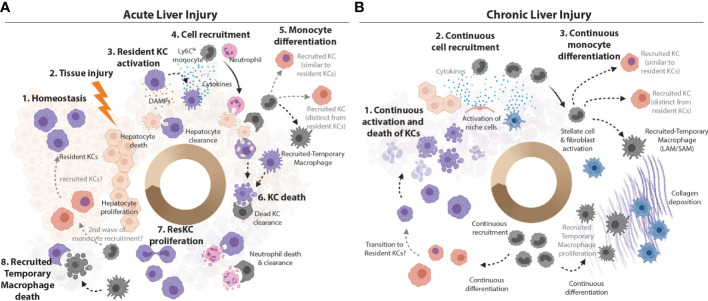
Plasticity *vs* Heterogeneity in the Inflamed Murine Liver. **(A)** KCs are the main macrophage population in the homeostatic liver. They are the sentinel cells that can sense PAMPs from attacking pathogens and DAMPs from dying hepatocytes in the first stages of infection or liver tissue injury. KCs can also efferocytose cellular debris from dead hepatocytes (47). Sensing of PAMPs and/or DAMPs activates KCs to produce chemokines and cytokines to call in monocytes and neutrophils from circulation (7). Ly6C^hi^ monocytes can differentiate locally depending on the cues from their microenvironment into recruited-temporary macrophages or KCs. However, recruited KCs have not been observed in all settings (15). Alongside the recruited neutrophils, these recruited macrophage populations may produce cytokines at the site, and help with cleaning up the pathogens/cellular debris. The loss of resident KCs has been reported in many inflammatory settings (4, 15, 23–25, 32, 38, 39, 48) and may be caused by stress resulting from activation or from the increased metabolic load of ingested cellular debris. Recruited cells such as recruited-temporary macrophages and neutrophils help to clean up the KC debris (49) and, in case of pathogen-induced inflammation, to capture/eliminate pathogens and pathogen infected cells. Notably, neutrophils have limited lifespan in the tissues (49) and their debris is subsequently also cleaned up by hepatic macrophages (33). With cellular debris removed, hepatocytes have space to replenish their numbers through proliferation. Resident KC numbers are also replenished through their proliferation and/or engraftment of recruited KCs which subsequently acquire the resident KC phenotype (TIM4 expression). However, exactly how similar resident and recruited KCs are when the latter are generated in an inflammatory environment remains to be seen. **(B)** If the inflammatory insult is not resolved (e.g., in case of chronic infection, repeated injury or metabolic stress associated with increased lipid burden), the resolution phase is not reached and chronic inflammation develops. Increased KC death activates the niche cells (LSECs, HSCs and hepatocytes) to call in Ly6C^hi^ monocytes to replenish lost KCs (4, 23–25, 32). Continuous activation and/or tissue injury may lead to death of structural cells of the liver. The recruitment of cells from circulation is continuous. Recruited monocytes differentiate locally into KCs and/or recruited-temporary macrophages, depending on the cues from their microenvironment. For example, activated HSCs produce and deposit increased amounts of collagen, which leads to liver fibrosis. Monocytes recruited to fibrotic zones harbouring large numbers of activated stellate cells/fibroblasts differentiate into recruited-temporary macrophages expressing CD9^+^TREM2^+^ called hepatic LAMs/SAMs (4, 30, 32). As with acute injury/infection/inflammation, recruited KCs can acquire the TIM4-expression with time (4, 23) and the cycle continues. As in acute inflammation, how similar or distinct recruited and resident KCs are remains a matter of debate.

Hepatic macrophages, initially considered to consist only of KCs were thought to be very plastic cells, being rapidly able to respond to any insult as required. However, now that we know there are in fact many distinct subsets of hepatic macrophages this has led us to question if this range of functions represents *bona fide* plasticity of resident KCs or if it rather reflects the heterogeneous nature of the hepatic macrophage pool in inflammation? This question is not unique to the liver, rather it can be asked of macrophages throughout the body. Indeed, the plasticity of the resident alveolar macrophages in the lung has also recently been questioned (18). However, while in that perspective it was proposed that tissue resident alveolar macrophages are not particularly plastic (18), as will be discussed below, evidence from distinct hepatic inflammatory insults would suggest this may not be the case for the resident KCs, where there appears to be an immediate, albeit acute, response (**Figure 2**). However, despite this initial response, we similarly postulate that the assumed hepatic macrophage plasticity observed later in the inflammatory response, is likely a function of heterogeneity within the hepatic macrophage pool, as recruited-temporary macrophages and to a lesser extent recruited KCs appear to respond differently compared with resident KCs. In addition, there is likely additional plasticity within these recruited populations as they appear to be able to edit their profiles according to the environment into which they are recruited (**Figure 2**). Compared with KCs, very little is known about capsule macrophages in infection/inflammation. Recently, these have been suggested to have a function in immune surveillance by sensing microbes in the peritoneal cavity and recruiting microbicidal neutrophils to the liver capsule (21). However, whether capsule macrophages are plastic and able to adapt to different environments has not yet been addressed. Thus, these cells will not be discussed further in this context. However, it is important to consider that these cells may be mixed with the recruited macrophage populations identified in many studies.

### Acute Plasticity of Resident KCs

The highly phagocytic nature of KCs and the plethora of complement and scavenging receptors on their surface (22, 26, 50) affords them the ability to rapidly identify and deal with threats entering the liver *via* the bloodstream (7, 51, 52). Moreover, through their interactions with LSECs, HSCs and hepatocytes (36, 37, 41) they have the potential to play a significant role in the orchestration of the hepatic response to injury, inflammation and damage. They can sense damage associated molecular patterns (DAMPs) released from dying or injured liver cells, while also communicating with these cells to instruct appropriate responses. Indeed, depletion of KCs from mice using the *Clec4f*-DTR mouse model rapidly activates HSCs and LSECs which produce TNFα and IL1β to recruit new monocytes to the liver to replace the depleted KCs (36). The presence of multiple subsets of macrophages and a lack of markers to discriminate them makes interpretation of KC-specific functions difficult for many studies. However, as resident KCs are the main population of macrophages present in the liver at the onset of injury, with recruited macrophages typically only appearing after 48 hours (based on expression of F4/80 and/or CD64 and lack of Ly6C expression) following their differentiation from monocytes, we can largely infer resident KC-specific responses within this time window.

Many studies have reported the production of cytokines and chemokines, as well as other factors such as complement factors and prostaglandins by KCs initially upon encountering inflammatory stimuli (7, 53–55). This suggests that resident KCs do retain some plasticity and can mount responses to inflammation and infection (**Figure 2**). In the setting of pathogen-induced inflammation, this is coupled with KCs encountering and engulfing pathogens or upon recognition of soluble pathogen products. Depletion of TLR4 from myeloid cells using *Lysm*-Cre mice significantly impaired phagocytosis and bacterial clearance in the caecal ligation and puncture model of polymicrobial sepsis (56), resulting in increased cytokine production in the liver, although whether this was from KCs is unclear. Suggesting the importance of resident KC clearance capacity, depletion of hepatic phagocytes using clodronate liposomes in mice infected with *Borrelia burgdorferi*, the pathogen causing Lyme disease in humans, facilitated the spread of bacteria resulting in increased bacterial burden in bladder, heart, joints and spleen (57). However, as KC depletion also activates the local niche cells (36), this altered bacterial load may result from an altered local liver environment. In sterile inflammation, the cue for this initial resident KC response is encountering DAMPs such as HMGB1 released from dying cells such as hepatocytes (47). However, phagocytosis of apoptotic bodies also plays a direct role in the initiation of cytokine production from resident KCs (58). *Fabp7* expression by resident KCs has been associated with this process as *Fabp7* KO mice demonstrated decreased phagocytosis of apoptotic cells and decreased cytokine production in acute liver injury (59). Similarly, clearance is suggested to be crucial in the context of viral infection. Ablation of KCs during acute viral hepatitis resulted in increased liver pathology and impaired apoptotic cell removal suggesting resident KCs may have protective effects (60). Although the potential caveats of KC depletion and niche activation also apply here. Conversely, treating HBV DNA carrying mice with an anti-PDL1 antibody reduced viral DNA persistence and favoured CD8 effector T cell differentiation indicating that the interaction between PD1 on T cells and PDL1 expressed by KCs might inhibit effective virus clearance (61). The production of cytokines, chemokines and other factors from resident KCs early after sensing the inflammatory stimuli is proposed to function in the recruitment of monocytes and neutrophils to the injured liver [reviewed in (7)]. Indeed, while resident KCs in the liver are specialized in sensing and capturing the infectious agent in the tissue, it is suggested that recruited monocyte-derived cells have potent microbicidal capacities (62, 63). Thus, this could explain the need for monocyte recruitment. Similarly, in sterile injury, monocyte and neutrophil recruitment is proposed to be important for repair. CCR2KO animals which have severely reduced monocyte numbers do not display any differences in terms of the extent of injury post APAP overdose, however, significant delays in tissue repair were observed (64). Furthermore, CCR2KO mice also have higher TNFα (mRNA) levels in the liver 24h after APAP (65), although this may arise from HSCs and LSECs attempting to recruit in monocytes (36). Importantly, repair post APAP overdose is further exacerbated when both resident KCs and monocytes/recruited macrophages are missing from the liver (66), suggesting that resident KCs and/or the niche cells could also directly contribute to repair. Apart from their ability to mount inflammatory responses, resident KCs have also been suggested to secrete tolerogenic mediators such as IL10 (55, 67, 68). It has been shown in a murine model of septic peritonitis that non-specific depletion of KCs by clodronate liposomes leads to a significant reduction in systemic and hepatic IL10 levels which was proposed to be primarily KC-derived (67). This correlated with a higher mortality, further suggesting that the initial resident KC response to inflammatory stimuli may be crucial. Taken together, this ability of resident KCs to respond to the changes in the local environment suggests that these cells do retain some plasticity despite having resided in the homeostatic liver for prolonged time periods before injury/infection.

While this resident KC response is relatively well documented, whether this response is protective or detrimental remains unclear. Possibly suggestive of a protective role for these cells, previous studies have shown increased serum AST/ALT levels indicating increased damage when resident KCs are depleted by clodronate liposomes 48 hours prior to administration of an APAP overdose (69, 70). Similarly, injury post APAP overdose (8 and 24 hours) is exacerbated in mice deficient in MerTK which harbour less KC at steady state (33). However, whether this worsened injury is linked to resident KC cytokine/chemokine production remains unclear. In one study, clodronate liposome treatment 48 hours prior to administration of APAP reduced TNFα, IL10, IL6, IL18BP and complement 1q mRNA levels in liver tissue 8 hours post overdose (69), however it should be noted that IL6, IL10 and C1q were already reduced prior to administration of APAP due to the depletion of resident KCs, while the baseline effects on TNFα were not assessed (69). The other study however, following the same treatment regime but administering higher concentrations of APAP, observed increased plasma IL6 and decreased IL1β levels 24 and 48 hours post overdose, while no effects were seen in plasma TNFα and IL10 levels (70). As clodronate liposome administration can also affect monocyte levels, either directly through their depletion (71) or indirectly by inducing monocyte recruitment to repopulate depleted macrophages, this is likely a confounding factor in this analysis, especially regarding cytokine production, as monocytes recruited to the liver may also produce these factors. Given that this enhanced monocyte recruitment upon resident KC depletion is also observed in KC-specific depletion models such as the *Clec4f*-DTR model (22, 36), we will need a more refined approach to assessing the function of KCs in inflammation rather than depletion. This is especially true, in light of the temporal niche activation observed post KC-depletion (36). The *Clec4f*-Cre model (50) should allow for a more specific interrogation of resident KC function if utilised prior to the development of any recruited KCs in inflammatory settings, alternatively an inducible *Clec4f*-Cre model would allow KC activation to be studied more accurately. In addition, it will also be necessary to consider heterogeneity in the resident KC response. Recently, scRNA-seq analysis identified activated resident KCs 20 hours after APAP or Thioacetamide (TAA) administration. Fitting with the above, these activated resident KCs were characterised by increased chemokine expression (72). Surprisingly, not all KCs in treated livers were activated, rather these cells represented a proportion of the total resident KC pool (72). The precise factors influencing this heterogeneity, including activation of the local niche, remain to be investigated.

### Lack of Long-Term Resident KC Plasticity in Acute Injury

While there appears to be evidence for plasticity early in the inflammation cascade, allowing resident KCs to respond and call in the required back-up, there is emerging evidence that this response may be tightly regulated in time. For example, while a subset of activated KCs was identified 20 hours after APAP overdose (72), bulk RNA sequencing data of KCs 72 hours after APAP overdose, a timepoint when there are significant numbers of recruited-temporary macrophages present in the liver, did not reveal any overt changes in the KC transcriptional profile (15). This could suggest that in this setting of sterile inflammation, KCs do not exhibit long-term plasticity. Alternatively, as scRNA-seq analysis demonstrated that it was only a subset of resident KCs that were activated at the early timepoint post APAP overdose (72), it is possible that this subset may have been overlooked in the bulk RNA sequencing data. Another possibility is that activated resident KCs may die shortly after their activation (**Figure 2**). This would fit with the reported reduction in KCs in sterile and pathogen induced inflammation (4, 15, 23–25, 32, 39, 48). Indeed, necroptosis of KCs in the early phase of infection such as seen with *Listeria monocytogenes* (Lm) is proposed to be an altruistic mechanism to attract cells with potent microbicidal functions (62). This mechanism appears to be conserved among macrophages in different tissues (3, 43, 73, 74) and thus might represent an additional way to recruit cells which are specialized in eliminating microbes such as neutrophils and monocytes. Thus, perhaps by 72 hours post APAP overdose the activated resident KCs are lost and hence the remaining population are those not activated initially. It is also important to note that due to the acute nature of the APAP overdose model, 72 hours post overdose the liver has already undergone considerable repair, thus the limited response of the KCs at this timepoint may be due to the absence of further hepatocyte death and associated signals to which a response from KCs is warranted. Considering this, it will be important to assess the temporal nature of the resident KC response following APAP overdose by profiling the cells at intermediate timepoints between 20 and 72 hours. If activated resident KCs are lost following APAP overdose at later timepoints, this opens up many questions including how quickly do the activated resident KCs die? Is their death necessary, for example is it important for priming the incoming monocytes? Moreover, what signals promote their death? Uptake of erythrocytes has been shown to lead to resident KC death (40), thus does efferocytosis/phagocytosis play a role here? Or could it be that an activated resident KC is no longer adapted to its environment and hence cannot be maintained? This assumption would demonstrate a lack of plasticity in these cells. Similarly, if resident KC plasticity is limited, perhaps it is the stress associated with this early activation that results in their death? Answering these questions will be crucial for our understanding of this response and how it can be manipulated for therapeutic benefit.

### Lack of Long-Term Resident KC Plasticity in Chronic Injury

While understanding the longevity of the resident KC response to inflammatory stimuli is limited in models of acute injury, recent studies investigating hepatic macrophage heterogeneity in the setting of more chronic inflammation such as MAFLD and non-alcoholic steatohepatitis (NASH) have shed further light on this. Fitting with a limited activation of resident KCs, we recently found that even after prolonged exposure to western diet (24-36 weeks) and establishment of MAFLD and NASH, resident TIM4^+^ KCs did not dramatically alter their transcriptional profile, particularly in relation to expression of prototypical immune activation genes such as pro-inflammatory cytokines (4). This was consistent with another study which did not see significant activation of total human hepatic macrophages from lean and obese patients or in total murine hepatic macrophages fed a high fat diet for 3, 9 or 12 weeks (14). Notably, in this study, an increase in *Tnf* was reported after 12 weeks of diet but here the macrophage population was not split into distinct subsets and this timepoint also correlated with the infiltration of recruited macrophages. Thus, this increase was attributed to the recruited macrophages (14). Conversely, however, other studies have reported changes in the resident KC transcriptome in murine models of NASH. While a first study likely mixed resident KCs and recruited-temporary macrophages (LAMs) leading to the conclusion that KCs were activated in NASH (75), another recent study where NASH was induced in mice by feeding a methionine and choline deficient diet (MCD diet), did report increased expression of pro-inflammatory genes in resident KCs after 6 weeks of feeding (23). Notably, here the authors did distinguish between KCs resident in the liver before and after NASH induction using BM chimeras demonstrating that those present before inflammation can be activated. Whether this is true of the whole population or a subset of resident KCs was not examined (23). Another recent study also found significant changes in the resident KC transcriptome upon feeding a NASH-inducing diet for up to 30 weeks (24). However, here the majority of these changes were associated with the loss of KC identity driven by altered LXR signalling (24), as observed when LXRα expression is removed from homeostatic KCs (50). This loss of KC identity may result from changes to the local microenvironment altering the signals given to the KC population (24). Another study found resident KCs to express reduced levels of Calprotectin encoded by *S100a8*/*S100a9* following 16 weeks of feeding a Western diet (76). These discrepancies regarding the resident KC response in MAFLD and NASH between studies highlight that we do not yet fully understand the mechanisms governing this. One possibility is that the diet used to induce NASH may alter the resident KC response as all studies utilised different diets for different periods of time. Nevertheless, this would further suggest some plasticity of the resident KCs, albeit dependent on the signals received.

Despite the differences in the degree of resident KC activation reported, all these NASH studies observed a reduction in the proportion and/or number of resident KC populations (4, 23–25, 32). This death and loss of resident KCs in NASH would fit with the concept that upon activation KCs can no longer self-maintain and are lost from the tissue as discussed above. This loss may also help to explain the heterogeneous response of resident KCs across models. For example, it is possible that activated resident KCs are not fit enough to survive the lengthy digestion processes used in some studies used to liberate these cells (4). The advent of techniques for profiling these cells without the need for tissue digestion such as snRNA-seq and spatial transcriptomics approaches will enable this question to be addressed in the near future and is something we are currently investigating. Alternatively, as suggested by the Glass lab, this loss of resident KCs may be irrespective of activation state but rather due to a loss of KC identity (24). Whether related to activation or identity, this loss of resident KCs suggests that despite maintaining some plasticity to respond initially, these cells are not plastic enough to adapt to the inflammatory environment long-term. Fitting with this, it would be very interesting to profile the resident KCs when the diets are first switched to assess if the change in diet has any effects on the resident KC population not observed after prolonged feeding.

### A Possible Role for the Microbiota in KC Plasticity

The specific microbiota composition of the mouse colonies in the different animal houses may also play a role in the distinct responses of resident KCs across models. Indeed, as KCs are preferentially localized in peri-portal zones, where they can more effectively capture and fight microbes coming from the intestine *via* the portal vein (37), differences in microbiota could explain these differential KC responses. Such modulation of the extent of liver injury by the microbiota has been reported in the APAP overdose model, where injury was attenuated in germ-free (GF) mice, or in SPF mice treated with antibiotics (72). The lack of microbiota inhibited the MYC-activated transcriptional program in various hepatic cell populations, leading to lesser injury (72). Fitting with a potential role for the microbiota, gut microbiota dysbiosis due to chronic use of antibiotics or proton pump inhibitors was implicated in higher susceptibility to acute liver failure after APAP overdose in humans, which was recapitulated in dysbiotic *Nlrp6^-/-^* mice and in WT mice that were transferred faecal microbiota from *Nlrp6^-/-^* mice (77). The use of antibiotics removed the difference in liver injury between WT and *Nlrp6^-/-^* mice (77), suggesting that a specific microbial species present in the *Nlrp6^-/-^* mice might have a negative effect on liver injury after APAP overdose. This effect could be mediated through altered microbial metabolites. For example, one such metabolite, 1-phenyl-1,2-propanedione has been shown to aggravate APAP-induced liver injury by modulating glutathione levels in the liver (78). Another study suggesting a role for commensal microbiota in shaping hepatic macrophage responses, has demonstrated that the absence of commensal microbes in GF mice resulted in an impaired ability of KCs to clear *Staphylococcus aureus* from circulation resulting in increased systemic pathogen load and higher mortality of these mice compared with mice kept in SPF conditions (79). In addition to regulating KC function, the microbiota has also been shown to affect the development of capsule macrophages. While sparse during neonatal development, the number of capsule macrophages increases significantly after weaning and is then maintained throughout adult life (21). However, whether the microbiota influences capsule macrophage plasticity in the liver remains to be investigated.

### Plasticity of Recruited KCs – A Role for the Local Environment?

As discussed above, as many studies have not yet accurately distinguished between hepatic macrophage populations, it is not possible yet to know if recruited monocytes always give rise to KCs in inflammatory settings or how plastic these cells may therefore be. Here we will discuss what is currently known or can be extrapolated from existing data. A key question when discussing this is how similar are these recruited KCs to their resident counterparts? Our own studies investigating newly recruited KCs following resident KC depletion under homeostatic conditions using the *Clec4f*-DTR mouse, did not identify any long-term differences in terms of the transcriptome or phagocytic capacity of the new recruits compared with resident KCs (22). We did not however, investigate any potential epigenetic differences nor did we probe their functionality in different inflammation models, thus we cannot rule out the possibility that recruited KCs may behave differently to resident KCs in some settings (**Figure 2**). Indeed, using BM chimeric mice, Beattie et al., have demonstrated that in mice treated with heat-killed pathogenic bacteria congenically-marked recruited KCs, generated following whole-body irradiation (resulting in KC depletion), exhibited an increased phagocytic potential compared to resident KCs (26). However, if resident KC function may have been affected by the irradiation is unknown. Conversely, a study by David et al., which used clodronate liposome to non-specifically deplete resident KCs has suggested that monocytes and recruited KCs have a reduced ability to phagocytose intravenously transferred non-pathogenic *E. coli* as evidenced by increased bacteria counts in the blood (80). Whether these discrepancies are due to differences in the system used to induce the differentiation of recruited monocytes into KCs or due to strain-specific features and/or viability of the different bacteria used remains to be determined. These studies could suggest that the specific setting in which KCs are generated may alter their behaviour. While at first sight this may suggest some plasticity, we would question if this could be considered as recruited KC plasticity or rather plasticity within the infiltrating monocytes. The effect of the KC-ablation protocol on the local microenvironment is also worth considering in these studies, as this may also have an impact on the fate of the infiltrating cells. Fitting with this, a recent study by Louwe and colleagues has demonstrated that the local environment coupled with the presence of any remaining resident macrophages appears to dictate the phenotype of recruited macrophages in the peritoneal cavity (12). In different inflammatory settings recruited peritoneal macrophages were all able to persist long-term, however, these were maintained in an immature transitory state when resident macrophages were also present but a mature resident-like state when the original resident macrophages were fully depleted (12).

### Plasticity of Recruited KCs – NASH

Differences between recruited and resident KCs have also recently been reported in the context of NASH. While recruited KCs have been described in a number of different studies irrespective of the precise dietary model used to induce NASH, how similar these are to their resident counterparts appears to be diet-specific (4, 23, 24). Fitting with the relative quiescence of the resident KC population observed after feeding a western diet for up to 36 weeks compared with controls, we did not observe significant differences in the transcriptional profiles of resident and recruited KCs in this model (4). Similarly, the study from the Glass lab did not observe significant differences between resident and recruited KCs in NASH (24). Conversely, the study from the Gautier lab, the only one to truly distinguish between recruited and resident KCs using BM chimeras rather than relying on the temporal lack of TIM4 expression, identified differences between resident and recruited KCs. Here, recruited KCs in the MCD diet model of NASH were more pro-inflammatory and less able to store triglycerides than their resident counterparts (23). Notably, increasing the recruited KC population in this model also led to exacerbated disease (23), although whether this is directly linked to the presence of recruited KCs or a result of an activated niche upon depletion of resident KCs to induce KC recruitment remains to be dissected. Interestingly, upon return to normal chow following feeding the MCD diet, recruited KCs were found to persist for at least 12 weeks (23). A previous study investigating recruited KC persistence in the MCD model of NASH using TIM4 expression to discriminate between resident and recruited KCs, found that TIM4^-^ recruited KCs were reduced following return to normal chow (25). Thus, this likely suggests that recruited KC persistence following recovery from NASH is associated with acquisition of TIM4 expression. This would also fit with BM chimera studies demonstrating no additional input from the BM to the hepatic macrophage pool post recovery, while a low level of chimerism was maintained in TIM4^+^ KCs (25). The key question now, is whether these persistent recruited KCs maintain their pro-inflammatory phenotype or if with time in the recovered liver they can alter their phenotype in line with homeostatic resident KCs as has been observed for monocyte-derived alveolar macrophages (3). Similarly, it will be intriguing to determine if the remaining resident KCs in this MCD-model also maintain their altered profile or revert to their homeostatic profile during recovery. Understanding this from both a transcriptomic and epigenetic viewpoint will provide valuable insight into the question of resident and recruited KC plasticity.

### Plasticity of Recruited KCs – Pathogen Induced Inflammation

What about recruited KCs in pathogen-induced inflammation? Infection with the facultative intracellular pathogen Lm has been shown to lead to a contraction of the resident KC pool followed by the recruitment of monocytes and the development of monocyte-derived macrophages in the liver (48). Based on the expression of F4/80, Ly6C and CD11b the authors of this study discriminate between resident KCs (F4/80^+^ CD11b^lo^ Ly6C^lo^), recruited macrophages (F4/80^+^ CD11b^hi^ Ly6C^int^) and monocytes (F4/80^+^ CD11b^int^ Ly6C^hi^) and proposed that both the recruited macrophages and resident KCs, proliferate in response to Lm infection in order to repopulate the available niches in the liver (48) (**Figure 2**). However, based on these markers, the precise fate of the recruited macrophages in this setting is unclear. Firstly, the definition as Ly6C intermediate raises some questions regarding their designation as macrophages which typically lack Ly6C expression. Next, it is unclear, if these resemble recruited-temporary macrophages or recruited KCs? In the setting of erythrocyte clearance, recruited macrophages were found to have the same F4/80 and CD11b expression profile as resident KCs (40), but even then, without KC-specific markers it is difficult to determine the exact nature of these cells. If the macrophages recruited in Lm infection become recruited KCs are these retained in the tissue post pathogen clearance or are these also lost being replaced by proliferating resident KCs? Notably, while Lm induced a type 1-dominated response characterized by increased *Ifng* transcripts in Ly6C^hi^ monocytes and recruited macrophages early after infection, genes associated with a tissue repair profile including *Arg1* and *Chil3* were upregulated in these populations during the resolution phase of the infection (48). This could suggest that these recruited monocytes and macrophages unlike the resident KCs are plastic being able to alter their phenotype as required by the tissue, starting out as an inflammatory monocyte and gradually differentiating into a repair macrophage. Alternatively, it could suggest that there are two waves of monocyte recruitment and macrophage differentiation and this remains to be investigated (**Figure 2**). Similarly, F4/80^+^ CD11b^lo^ Ly6C^lo^ cells defined in this study as resident KCs showed increased *Chil3* transcript levels at later stages of the infection (day 3) (48), suggesting the resident KCs may have additional plasticity in this model. However, another likely option is that at this timepoint recruited macrophages (KCs and/or recruited-temporary macrophages) can be found within the cells defined here as resident KCs. In this setting, one hypothesis could be that the *Chil3* expression is restricted to recruited macrophages infiltrating this gate. With this possibility in mind, it would be interesting to evaluate the nature of the recruited macrophage population and determine how long these populations and the *Chil3* signature persists post infection.

Consistent with Lm infection, infection with Vaccina virus has been shown to lead to an almost complete loss of resident KCs which was also compensated by the recruitment of monocytes (81). A similar depletion of resident KCs was also observed with murine cytomegalovirus infection (81) further highlighting the conserved nature of this response. Using CLEC4F expression as a marker of *bona fide* KCs, Borst et al. were able to demonstrate that the recruited monocytes in this instance subsequently differentiated into KCs (81) (**Figure 2**). Notably, in vaccinia infection, recruited KC development was regulated through type I IFN and IFNAR signalling. In a competitive BM chimera setting, IFNAR KO monocytes more efficiently engrafted and differentiated into recruited KCs than WT counterparts (81). Loss of IFNAR signalling also altered the recruited KC phenotype, increasing expression *Arg1, Ym1* and *Nos2* (81). This nicely suggests that recruited KCs do display some plasticity as they respond differently depending on the cues they receive from the local environment. Alternatively, as IFNAR expression was eliminated from either all cells (full body KO) or LysM expressing cells, this altered phenotype of recruited KCs may instead be a feature of monocyte and not recruited KC plasticity.

During the reproductive cycle of the blood fluke *Schistosoma mansoni* in vertebrate hosts some eggs are deposited in the liver leading to a type 2-driven granulomatous reaction (82, 83). During this process IL4/IL13 alternatively activated hepatic macrophages accumulate around granulomas promoting tissue repair and the formation of fibrotic tissue (84). Rolot et al. have recently demonstrated that infection with *S. mansoni* resulted in a strong reduction of the resident KC pool, accompanied by an increase in CD64^+^F4/80^+^CD11b^hi^ recruited macrophages (39). This increase in CD11b^hi^ macrophages was independent of IL4Ra (39), which is in contrast to serous cavity macrophages (resident and recruited) which exhibit strong, IL4-dependent proliferation in response to nematode infection (85, 86). Through the use of shielded BM chimeras, these CD11b^hi^ macrophages were demonstrated to be of monocyte origin (39). This observation is in line with previous reports demonstrating that increased numbers of monocyte-derived macrophages can be found in the liver after *S. mansoni* infection (87, 88). While like in the other infection models, it is tempting to speculate that these may represent recruited KCs, as KC-identity gene expression was not assessed, the specific nature of these cells remains to be determined. Notably, these cells were found to proliferate suggesting they are capable of self-renewal (**Figure 2**), although it is not clear if there is also continual replenishment from monocytes over the course of the infection (10 weeks) (39). In comparison, the remaining few resident KCs showed limited proliferative capacity potentially explaining their loss over the course of the infection (39). In terms of the phenotype, the recruited macrophages, in addition to their increased expression of CD11b, also expressed *Arg1, Chil3, Retlna* and *Nos2* compared with resident KCs (39). This phenotype was dependent on IL4Ra signalling, although loss of IL4Ra from myeloid cells did not alter host survival post infection (39). This limited response of the resident KCs compared with the recruited macrophages again highlights the plasticity of incoming monocytes and/or differentiating macrophages compared with resident KCs.

Taken together, while we do not yet have all the information, recruited KCs appear to be capable of editing their phenotype based on their local environment. However, whether this represents recruited KC or monocyte plasticity remains to be directly tested. Moreover, whether these cells are really more plastic than resident KCs remains to be formally examined although evidence discussed above would suggest this is the case. It would be interesting to examine how similar or distinct the recruited KC profile is across models of inflammation to determine exactly how plastic these cells are. With the recent boom in scRNA-seq data, such a comparison may be possible relatively soon. Moreover, it will be important to determine the fate and plasticity of these cells upon elimination of the inflammatory stimuli. Do these cells always persist and if so, how similar are their transcriptomic and epigenetic profiles during and after resolution of inflammation? Finally, it will also be crucial to assess how efficiently these recruited KCs, generated in the course of inflammation can respond to additional stimuli.

### Recruited-Temporary Macrophages-NASH

Rather than differentiating into KCs, monocytes recruited to the inflamed liver may also take on an alternative fate and differentiate into recruited-temporary macrophages (**Figure 2**). The factors controlling this decision remain to be determined, however, as is the case for the KCs, this is likely decided by the local micro-environment into which the monocytes are recruited, due to local cell-cell interactions or possibly local metabolite concentrations. For example, in the setting of MAFLD and NASH, monocytes can differentiate into either KCs or Lipid-associated macrophages (LAMs; **Figure 2**) (4, 32). LAMs, and their putative precursors, called c-LAMs, preferentially localise in zones of fibrosis (**Figure 2**), while recruited KCs were found to be located in similar zones to their resident counterparts (4, 32). This localisation of LAMs suggests that the local signals provided by fibrotic stellate cells and other activated fibroblasts may contribute to the differentiation of these cells, although the precise signals involved are currently unknown (**Figure 2**). In addition to signals deriving from the activated fibroblasts in NASH, signals from dead/dying hepatocytes may also be involved in instructing the LAM/c-LAM phenotype as c-LAMs have been shown to be specifically enriched in hepatic crown-like structures (hCLS) (32) which are specific histological formations consisting of macrophages surrounding lipid-rich apoptotic hepatocytes characteristic of NASH (89). This is not the first description of hCLS-macrophages in the fatty liver, as a population of CD11c^+^ macrophages have also been described around these structures in the MC4R KO model of NASH (90). There, they were proposed to arise from resident KCs, however given the expression of *Itgax* (encoding CD11c) by LAMs/c-LAMs and lack of expression by KCs (32), it is tempting to speculate that these also represent recruited-temporary macrophages. However, as the two studies use distinct models of NASH, this also need further investigation. Finally, local lipid exposure could also play a role in driving the LAM phenotype, since a similar population of recruited-temporary macrophages are also observed in obese adipose tissue (91).

In fibrotic and cirrhotic human livers, a population of recruited macrophages called scar-associated macrophages or SAMs have been described in fibrotic zones (30). Human SAMs and murine LAMs display very similar phenotypes and transcriptional signatures (4) highlighting the potential clinical relevance of recruited-temporary macrophages. Notably, the temporary nature of these macrophages has not been demonstrated in mice or humans as no studies have yet investigated their persistence post recovery. However, we would hypothesize that if the fibrotic niche of these cells would be lost, then the LAMs would also fail to thrive. An alternative hypothesis could be that upon resolution, LAMs would alter their phenotype to become KCs. This outcome would depend on exactly how plastic these cells are following their differentiation and existence in the fatty liver and hence investigating this represents an interesting goal. Another outstanding question regarding these cells relates to their origins. Is it the same monocyte precursor that gives rise to both KCs and LAMs/SAMs or are there subsets of monocytes in the BM, possibly induced by the systemic inflammation that have a restricted differentiation potential? A population of pro-fibrotic monocytes has been described in the setting of lung fibrosis that are critical for the development of fibrosis (92) and thus investigating if hepatic macrophage heterogeneity stems from monocyte heterogeneity represents an interesting question for the future.

### Recruited-Temporary Macrophages-Acute Sterile Injury

What about other models of inflammation, are recruited-temporary macrophages generated and how distinct are these cells across inflammatory settings? Following APAP overdose, a population of recruited-temporary macrophages have been described. These cells differentiate from monocytes between 48 and 72 hours post overdose and are lost from the tissue upon resolution of inflammation between 5 and 7 days post the overdose (15). Notably, no recruited KCs have been reported following APAP overdose. This is despite the fact that a reduction in the resident KC pool (proportion and number) has been reported in this model suggesting the niche is available (15, 38). The precise reasons for the lack of recruited KCs thus remains to be investigated, however, given the strict zonation of the injury around the central vein, a zone usually largely devoid of KCs it is possible that the niche in that location is not permissive of KC generation. To assess this, it will be important to investigate the location of recruited monocytes and the signals present in their local environment skewing monocyte differentiation. An examination of the recruited-temporary macrophages 72 hours post APAP overdose demonstrated that they have a very distinct transcriptional profile to KCs with 135 differentially expressed genes (DEGs). Note only 2 DEGs were described between homeostatic and APAP KCs at this timepoint (15). In addition to expressing genes associated with a monocyte origin including *Ccr2* and *Cx3cr1*, APAP recruited-temporary macrophages also expressed many restorative genes including *Mmp8, 14* and *19* and extracellular matrix structural components including *Thbs1*, *Fn1* and *Vcan*, suggesting these cells may play distinct roles to KCs in repair. However, as many of these genes were shared with Ly6C^hi^ monocytes the specific roles of these cells remain to be seen. Similar to the mouse, human APAP overdose is also characterised by an increase in macrophages (33). These were found to express MerTK and CD163 and were localised around the central vein, where injury occurs (33). As relatively few MerTK^+^ macrophages were found in the liver outside of these zones (33), this could suggest these cells are the human recruited-temporary macrophage equivalents (**Figure 1B**). In both mouse and human, how similar these APAP recruited-temporary macrophages are to those in other inflammatory settings has not yet been investigated. In NASH, the recruited-temporary macrophages were termed LAMs due to their similarity with LAMs described in obese adipose tissue (91). As APAP overdose is not associated with increased dietary lipid content and obesity, it is tempting to speculate that the recruited-temporary macrophages here will have a distinct phenotype to LAMs. However, as recruited-temporary macrophages likely function to clear up cellular debris including lipid-material (**Figure 2**), perhaps these cells will have a similar profile across models.

### Recruited-Temporary Macrophages-Pathogen-Induced Inflammation

In the context of infection, the nature of recruited-temporary macrophages is less clear. As described above, infection is associated with loss of resident KCs and recruitment of monocytes which differentiate into macrophages. For the most part, whether these recruited macrophages represent KCs or recruited-temporary macrophages remains to be investigated (**Figure 2**). Along these lines it is also possible that both recruited-temporary macrophages and recruited KCs may be present in these settings, however, to date as these cells have not been distinguished it is impossible to speculate on how plastic and distinct these populations may be. Understanding this will be a key goal for the field in the coming years.

### Recruited-Temporary Macrophages: The Cause of the Perceived Plasticity?

Overall, given the relative paucity of studies discriminating recruited macrophage populations in different inflammatory settings, it is more difficult to assess the responses and associated plasticity of these cells and hence further studies are needed to dissect this. It is, however, worth mentioning that in the context of MAFLD and NASH, the LAMs displayed distinct transcriptional and lipidomic profiles from resident and recruited KCs suggesting that these cells are quite distinct from KCs (4, 32). Similarly, recruited-temporary macrophages in APAP are quite distinct from KCs (15). However, whether these cells are more plastic than KCs is unclear. Moreover, any plasticity in this population could reflect the limited time spent in the tissue and the high plasticity of the monocytes giving rise to these cells. If recruited-temporary macrophages were truly more plastic after their development, one might argue that these cells would not be ‘temporary’ and lost from the tissue upon resolution of inflammation. Rather one might expect that these cells would be able to adjust to the non-inflamed environment enabling them to be maintained in the liver upon recovery. Notably, in the few cases where recovery has been assessed to date such as following APAP overdose, this does not seem to be the case. Of course, it could also be argued that this lack of residence of the recruited-temporary macrophages is not reflective of a lack of plasticity but rather informative of a lack of an available niche in which they can be maintained (20). To test this, KCs could be specifically depleted in the initial phases of resolution prior to loss of (CLEC4F^-^) recruited-temporary macrophages. If truly plastic, we would hypothesise that the recruited-temporary macrophages may then alter their phenotype to become KCs and fill at least some of the available KC niche. If not plastic, a new wave of monocytes would be expected to engraft and repopulate the empty KC niche. Although seemingly theoretical, understanding the plasticity of recruited macrophages may be instrumental in our ability to target these cells for therapeutic purposes. The presence of recruited macrophages across inflammatory models, suggests that these could be a useful population to target clinically. Developing our understanding of these cells in the context of liver inflammation thus represents an important aim for the future.

## Concluding Remarks and Future Perspectives

While in homeostatic conditions resident KCs and capsule macrophages can be identified in the liver, in recent years numerous studies have provided compelling evidence that in inflammatory conditions there is considerably more heterogeneity among hepatic macrophages. Depending on the nature of the inflammatory stimulus and the timepoint during/after the insult recruited KCs and/or recruited-temporary macrophages can be identified in the liver in addition to the aforementioned populations. As this heterogeneity leads us to question the assumption that KCs are highly plastic macrophages, here we have discussed what is currently known regarding the different responses of the distinct hepatic macrophage subsets. It is clear from this discussion, that we still have a long way to go before we understand this fully. While we have started to gather some evidence, which clearly suggests that recruitment of macrophages in inflammation plays a significant role in explaining the perceived plasticity of the global hepatic macrophage population, a number of questions remain. Specifically, is the loss of KCs in inflammation caused by a lack of plasticity or rather is it an altruistic mechanism to recruit cells with specialized functions? Are resident KCs capable of responding long-term in the context of chronic inflammation, or is this response dampened upon recruitment of monocyte-derived macrophages? With regards to the recruited macrophages, while these indeed appear to be more plastic than the resident KCs, questions remain regarding whether this increased plasticity is observed in the monocyte or differentiated macrophage and if it is linked to ontogeny or the time they spend in the tissue? Moreover, whether this increased plasticity is observed following resolution of inflammation remains to be tested in the liver. Finally, it will also be crucial to better understand the signals provided by the liver niche and their impact on the development of the different macrophage populations and the maintenance or loss of their plasticity. We anticipate that in the coming years the increased availability of classical and spatial transcriptomic technologies and the development of specific tools allowing the distinct macrophage populations to be fate-mapped and/or targeted will considerably advance our understanding of macrophage heterogeneity, functions and plasticity in the liver. Using these tools and technologies to gain a better understanding of hepatic macrophage biology in mice and humans will allow to develop new and/or refine existing strategies to target these cells therapeutically.

## Author Contributions

CZ, AB, and CS wrote the manuscript and designed the figures. CZ and AB contributed equally to the manuscript. All authors contributed to the article and approved the submitted version.

## Funding

CZ is funded by a Marie Curie IntraEuropean Fellowship. (Mactivate #101027317) CLS is a Francqui Professor and her lab is funded by an ERC starting grant (MyeFattyLiver #851908), a UGent BOF starting grant and an FWO Project Grant (3G000519).

## Conflict of Interest

The authors declare that the research was conducted in the absence of any commercial or financial relationships that could be construed as a potential conflict of interest.
